# In Vitro Selection of a Single-Stranded DNA Molecular Recognition Element against the Pesticide Fipronil and Sensitive Detection in River Water

**DOI:** 10.3390/ijms19010085

**Published:** 2017-12-28

**Authors:** Ka L. Hong, Letha J. Sooter

**Affiliations:** 1 Department of Pharmaceutical Sciences, Nesbitt School of Pharmacy, Wilkes University, 84 W. South Street, Wilkes-Barre, PA 18766, USA; 2 Independent Researcher, Pittsburgh, PA 15234, USA; lethasooter@rocketmail.com

**Keywords:** SELEX, in vitro selection, aptamer, molecular recognition element (MRE), fipronil, pesticide, insecticide, ELISA, Decoy-SELEX

## Abstract

Fipronil is a commonly used insecticide that has been shown to have environmental and human health risks. The current standard methods of detection for fipronil and its metabolites, such as GC-MS, are time consuming and labor intensive. In this study, a variant of systematic evolution of ligands by exponential enrichment (SELEX), was utilized to identify the first single-stranded DNA (ssDNA) molecular recognition element (MRE) that binds to fipronil with high affinity (*K_d_* = 48 ± 8 nM). The selected MRE displayed low cross binding activity on various environmentally relevant, structurally unrelated herbicides and pesticides, in addition to broad-spectrum binding activity on major metabolites of fipronil and a structurally similar pesticide in prepared river samples. Additionally, a proof-of-principle fluorescent detection assay was developed by using the selected ssDNA MRE as a signal-reporting element, with a limit of detection of 105 nM in a prepared river water sample.

## 1. Introduction

Fipronil has been a widely used phenlpyrazole insecticide in the United States since it was first introduced in the late 1990s [1]. Fipronil inhibits gamma-aminobutyric acid (GABA) gated chloride channels and causes continuous excitation of the central nervous system, which ultimately leads to insect death [2]. Fipronil is often used as an alternative to organophosphate pesticides in many settings, including residential, commercial, and agricultural, due to its selectivity on insect GABA receptors [3]. There was a ten-fold increase in fipronil sales in California from 2000 to 2005 [4]. A report by Simon-Delso et al. indicates that fipronil currently accounts for approximately 10% of the global pesticide market [5].

Fipronil has become a widespread environmental contaminant because of its wide applications. In the United States from 2002 to 2011, fipronil was detected in urban streams up to 63% of the time, and 15 to 20% of the time in agricultural and mixed land [3]. Additionally, measured concentrations of fipronil exceed the aquatic-life benchmark in 70% of urban streams and more than 20% of agricultural and mixed land streams in this timeframe [3]. The chronic benchmarks for fish species and aquatic invertebrates are 6.6 µg/L and 9.8 µg/L, respectively [6]. It has also been shown to contaminate drinking water sources in Vietnam [7]. Worldwide exposure of humans and ecosystems to fipronil has been clearly observed.

The presence of fipronil in urban streams has been reported. It suggested that fipronil is highly toxic to many steam invertebrates, with a mean 96-h viability inhibition EC_50_ values as low as 32.5 ng/L for *Chironomus dilutus* [8]. In addition to the toxicity in aquatic invertebrates, fipronil is highly toxic to many fish species, with a reported 96-h LC_50_ level as low as 0.246 mg/L for rainbow trout and 0.083 mg/L for bluegill sunfish [1]. It also has a tremendous detrimental effect on non-target insects, such as honey-bees [9]. Although fipronil binds more selectively to insect GABA receptors than mammal GABA receptors, a long term toxicity study showed it increased thyroid follicular cell tumors in rats [10,11]. The U.S. Environmental Protection Agency has therefore classified fipronil as a possible human carcinogen [10]. Even though there has been an overall increase in the knowledge of how fipronil is impacting the environment and human health, a large knowledge gap still exists in terms of its long term environmental fate in various media, such as soils and agricultural products [12]. Thus, it is important to monitor the exposure levels of fipronil in the environment.

Currently, the detection of fipronil levels in environmental and biological samples are mostly dependent on chromatographic methods, such as gas and/or liquid chromatography coupled with mass spectrometry [13,14,15,16]. These methods are sensitive, but costly, and time and labor intensive. There have been reported uses of antibody-based enzyme-linked immunosorbent assays (ELISA) to detect total fipronil levels in human blood samples and artificially contaminated tap water samples [17,18,19]. However, there are inherent limitations in antibody-based assays. They are difficult or non-reusable, expensive to produce, and may suffer from batch-to-batch variations [20,21]. There is a clear need to develop a low-cost, rapid, reusable means to detect fipronil. One possible way to achieve this is by identifying a single-stranded DNA (ssDNA) molecular recognition element (MRE) that specifically binds to fipronil.

The process, systematic evolution of ligands by exponential enrichment (SELEX) can be utilized to identify such a specific binding element [22]. This in vitro selection process involves repeated cycles of incubation of a target with a library of up to 10^15^ different molecules, followed by partitioning of binders and non-binders under increasing selective pressure.

This study utilized a stringent variation of the SELEX process previously described by Hong et al. to select a ssDNA MRE with high binding affinity toward fipronil [23]. This Decoy-SELEX variant emphasizes on introducing multiple, lengthy negative selection rounds to direct the enriched library away from binding to structurally similar molecules and molecules that are likely to co-exist in the same environment [23,24,25,26]. In this study, major metabolites of fipronil, commonly used herbicides and pesticides (atrazine, malathion and propanil), and a closely related pesticide, ethiprole have been used as negative targets. Bovine serum albumin (BSA) was chosen to be one of the negative targets, as it is a common blocking agent. In addition, the selected ssDNA MRE was also incorporated into a proof-of-principle fluorescence assay for the detection of fipronil in prepared river water at nanomolar concentrations.

## 2. Results and Discussion

### 2.1. Identification of Fipronil Specific ssDNA MRE

**Figure 1:**
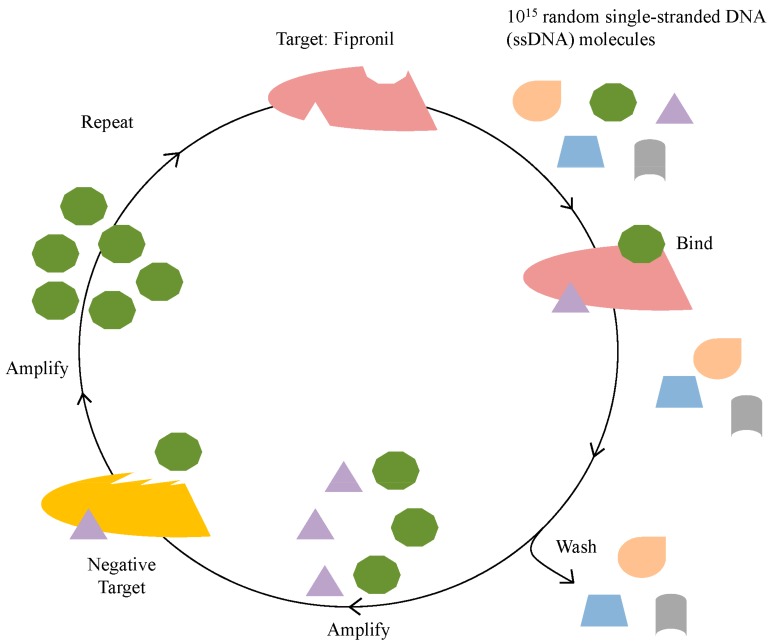
Illustration of the in vitro selection process. The in vitro selection process begins with up to 10^15^ different ssDNA molecules (represented by various small, colored shapes) and incubation with the target of interest, fipronil. Molecules that bind to fipronil are amplified and subjected to incubation with negative targets. Those that do not bind to negative targets are isolated and amplified. This completes one round of an in vitro selection cycle.

**Figure 2:**
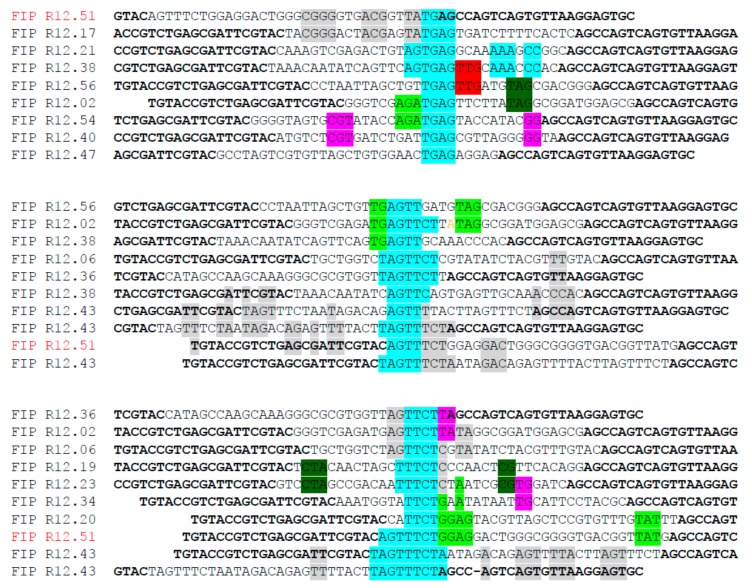
Representative sequence families of the round 12 library. Partial constant regions are shown in bold letters. The variable regions are shown in regular letters. R12.51 candidate MRE represented in multiple sequence families, and shared similarity with other sequences in their corresponding families. The turquoise color indicates the center for alignment in each family and other colors represent sequence similarity of each sequence.

Twelve rounds of SELEX were carried out to identify ssDNA MREs specific to fipronil (Figure 1, Table 1). Thirty eight, thirty three, thirty three, and thirty five of post round 3, 6, 9, and 12 library clones were sequenced and analyzed for consensus sequence families, respectively. Round 12 sequences were also subjected to Mfold analysis for secondary structures with associated Gibbs free energy values (ΔG) (indicating stability). One candidate sequence, designated R12.51 appeared to be highly conserved in multiple sequence families. The random region of R12. 51 shares 30%, 32% and 38% identity with the random regions of R12.43, R12.20 and R12.17 respectively (Figure 2).

**Figure 3:**
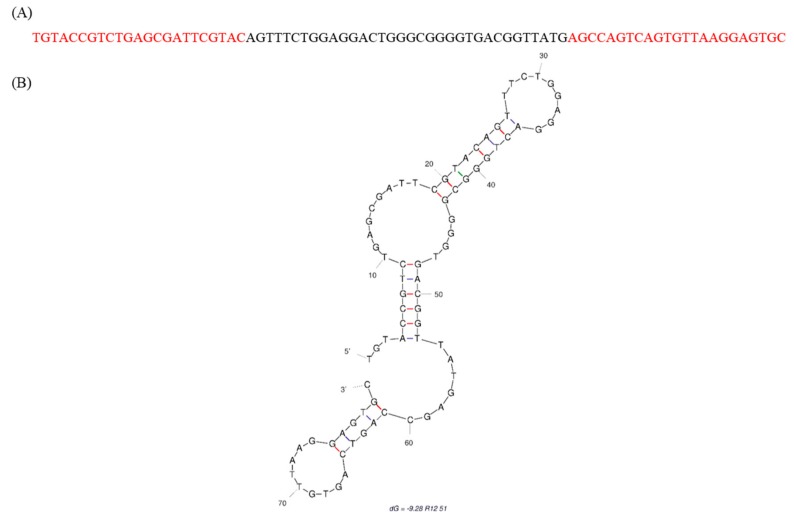
Secondary structure and sequence of R12.51 candidate ssDNA MRE. (**A**) ssDNA sequence of candidate fipronil MRE R12.51. The red portions indicate the constant regions for primer attachment, and the black portion indicates the variable region; (**B**) Mfold prediction of R12.51 secondary structure [27].

The Mfold DNA folding web-server predicts secondary structures of ssDNA sequences with given temperatures, sodium ion and magnesium ion concentrations [27]. The Mfold predicted stem–loop features of R12.51 were comprised of the random region and the constant regions, and had a Gibbs free energy of −9.28 kcal/mol (Figure 3). Previous studies demonstrated the constant region participates in binding events, and therefore may not be ignored when analyzing candidate MRE sequences and predicted secondary structures [28,29,30,31,32]. Based on these criteria, R12.51 was chosen as the candidate MRE for characterization studies.

### 2.2. Affinity and Specifcity of Fipronil-Specific ssDNA MRE in Selection Buffer and Buffered River Water

**Figure 4:**
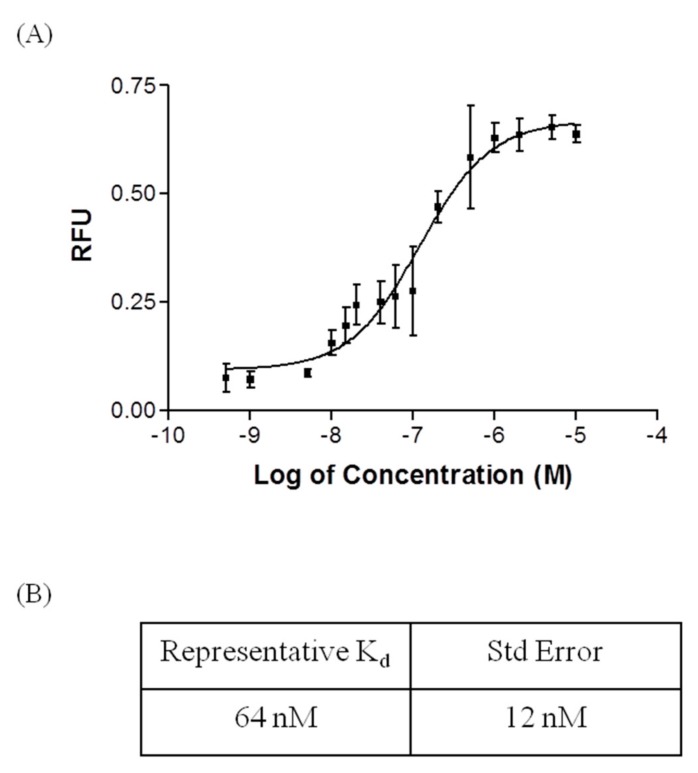
Fluorescent equilibrium binding assay of R12.51. (**A**) Representative saturation binding curve of R12.51 with nonlinear regression sigmoidal curve best fit. Concentration data was transformed by logarithmic scale. Normalized average fluorescence from triplicate samples were presented. Error bars are representative of ±1× standard deviations. The goodness of fit (*R*^2^) was 0.87; (**B**) Representative equilibrium dissociation constant with standard error for the presented R12.51 binding assay.

Four independent fluorescent saturation binding assays were conducted to determine the affinity of the R12.51 candidate sequence. Concentrations of the candidate sequence in the low nM to 10 µM range were assayed. The equilibrium dissociation constant (*K_d_*) from the four binding assays were 55 ± 14 nM, 35 ± 9 nM, 38 ± 12 nM and 64 ± 12 nM. The average equilibrium dissociation constant (*K_d_*) was 48 ± 8 nM (Figure 4). This plate-based fluorescence saturation binding assay method was modified from a previously reported bead-based assay [24,25,26]. The equilibrium dissociation constant obtained for R12.51 is comparable to other small molecule binding ssDNA MREs identified with other variants of SELEX [33,34,35]. This high affinity further validates the stringency of this SELEX scheme [36,37].

The cross-binding activity of R12.51 on negative targets used in the selection was also determined. This assay was modified from a previously described bead-based assay [24,25,26]. The data is presented relative to binding between R12.51 and fipronil as has been previously described [24,25,36,37]. The ssDNA MRE preferentially binds to fipronil, compared to atrazine (*p* = 0.030), propanil (*p* = 0.005), and malathion (*p* = 0.039) in buffer conditions (Table 2). The binding between fipronil, fipronil metabolites and ethiprole are not significantly different in buffer conditions. It is likely due to their high degrees of structural similarity. This suggests R12.51 potentially binds to the core chemical structure that is shared among fipronil, fipronil metabolites and ethiprole. A relatively high concentration of the negative targets (1 µM) were used in the selection buffer cross-binding assays, as the same concentration was introduced during the selection process (Table 1).

Interestingly, the cross-binding activity of R12.51 in a river water/buffer mix is noticeably different than in selection buffer alone (Table 3). A lower environmentally relevant concentration of negative targets was examined. The ssDNA MRE binds to fipronil significantly higher than it does to atrazine, propanil, and malathion in a river water/buffer mixed condition. While there are statistically significant differences in the binding selectivity ratios between fipronil, fipronil metabolites and ethiprole, the differences are small. It is to be noted that the river water/buffer mix alone did not elute a detectable level of R12.51 bound to immobilized fipronil. The difference between cross-binding profiles of selection buffer and the river water/ buffer mix is likely due to the water hardness found in the sampled river [38]. It is known that divalent cations such as Ca^2+^ and Mg^2+^ can greatly stabilize the secondary structures of nucleic acid MREs [39]. Hard-water containing a sufficient amount of these divalent cations potentially stabilizes the secondary structure of R12.51 when it binds to fipronil. The selectivity ratios between fipronil and structurally related compounds remain relatively unchanged in both binding conditions. This is again likely due to the close structural similarity between these compounds. It is to be noted that all the fipronil metabolites used in this study are environmentally relevant degradation products of fipronil [40]. This property of broad-spectrum binding to fipronil metabolites is desirable in an environmental biosensor.

Binding of R12.51 to BSA is strong in both binding conditions. This may be due to the large globular nature of the protein and its non-specific interaction with single-stranded DNA. This non-specific interaction appears to be difficult to reduce, despite of having two rounds of negative selection against BSA. High levels of non-specific binding between a small molecule specific ssDNA MRE and BSA was also observed in a previous study [26]. The same library utilized in this study, designated RMW.N34 has been studied in two other protein-targeted in vitro selection projects [36,37]. A phenomenon was observed from these studies in regards to the binding selectivity of the library molecules subjected to the Decoy-SELEX variant. It is possible that the binding selectivity of the library is enriched during early rounds of the selection process based on the molecular sizes of the target/or negative targets. In this study, BSA was first introduced after six rounds of enrichment. It is possible that the library was already enriched to a degree that a majority of the library molecules had non-specific binding to globular protein. This therefore suggests that early rounds of negative selection against globular proteins may be advantageous in magnetic bead-based small molecule SELEX schemes.

### 2.3. Biosensing Application of Fipronil-Specific MRE in River Water 

**Figure 5:**
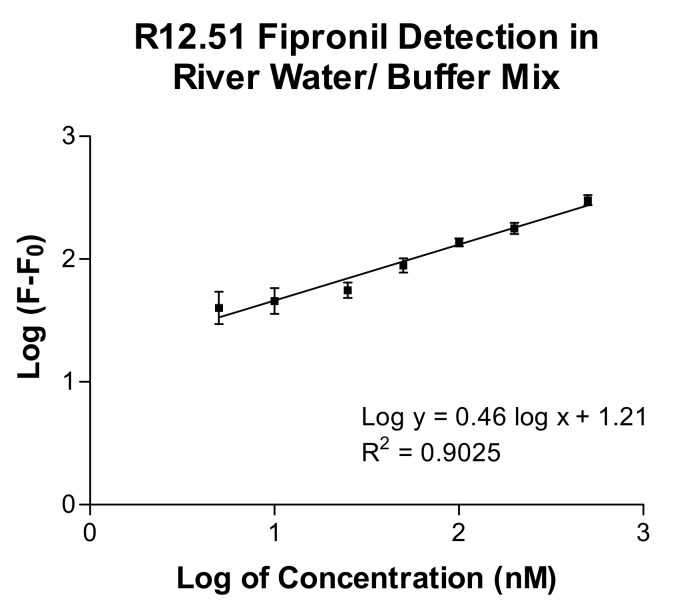
The calibration curve of fluorescence intensity with increasing concentrations of fipronil spiked in river water/buffer mix. Data is transformed by logarithmic scale. Normalized average fluorescence from triplicate samples are presented. F_0_ and F are values of normalized fluorescence intensities without and with fipronil. Buffer: 1× selection buffer. Error bars are representative of ±1× standard deviations.

The broad-spectrum binding profile of R12.51 and its high selectivity in conditioned river water allowed the investigation of its potential application as a biosensing tool. A plate-based biosensing platform using R12.51 as the reporter element was developed. Filtered river water/buffer mix was spiked to contain concentrations of 0, 5, 10, 25, 50, 100, 200, and 500 nM of fipronil. The spiked samples were incubated with FAM-labelled MRE bound to immobilized fipronil. The amount of MRE eluted with spiked sample was retrieved and the fluorescence signal was recorded on a plate reader. The calibration curve of fluorescence intensity and a linear correlation (*R*^2^ = 0.9025) between the value of normalized fluorescence and fipronil concentration were generated (Figure 5). The limit of detection (LOD) was calculated to be 105 nM based on the method previously described by Armbruster et al. [41].

There were two publications and one patent of fipronil detection immunoassays in the literature at the time this study was written [17,19,42]. In the study by Vasylieva et al., the author tested the cross reactivity of two candidate fipronil binding antibodies to structurally related compounds in complex biological and environmental matrices. Both candidate antibodies displayed a moderate to high levels of cross reactivity to fipronil metabolites, fipronil sulfide, fipronil sulfone, fipronil desulfinyl and ethiprole, that were also tested in the current study [19]. This showed that traditional antibodies also have a limited ability to differentiate the minor structural differences between fipronil and its metabolites. The reported IC_50_ values of the immunoassays were in the low microgram per liter range [19]. Although the LOD of the detection assay in this study was approximately ten-fold higher than environmentally relevant fipronil concentration, this may be enhanced with various signal enhancers, such as quantum dots, gold nanoparticles and horse radish peroxidase-conjugated ssDNA MREs in future biosensor development [43,44,45]. It is important to note that this study showed the identification of a fipronil binding element without the use of a hapten or animals. It also identified the first ssDNA MRE with high binding affinity to fipronil and structurally related compounds. One intrinsic limitation of R12.51 is its strong non-specific binding to BSA. This suggests additional sample preparation, such as push filtering, would be needed to remove protein contaminants that are likely to non-specifically bind to R12.51. In sum, the ability of candidate R12.51 to be applied in a proof-of-principle detection assay in environmental matrix has been demonstrated, which has the potential to be further developed into a ssDNA MRE based biosensor [46].

## 3. Materials and Methods 

### 3.1. In Vitro Selection for Fipronil-Specific MREs

**Figure 6:**
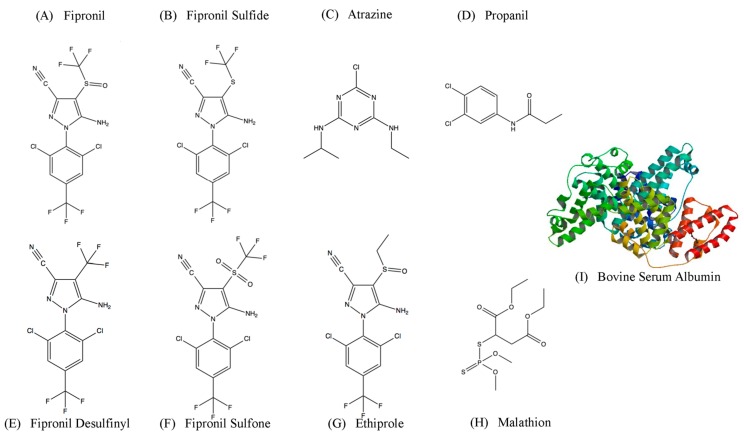
Chemical structures of targets used in the SELEX process and cross-binding assays. (**A**) Structure of the pesticide and target of selection fipronil; (**B**,**E**,**F**) Structures of fipronil metabolite; (**G**) Structures of ethiprole: chemically similar to fipronil; (**C**,**D**,**H**) Structures of atrazine, propanil and malathion: herbicides and pesticides commonly found in similar environments as fipronil. (**I**) Bovine serum albumin (PDB 4F5S, 66.5 kDa) [47].

In brief, the selection began with a ssDNA library with up to 10^15^ different molecules. This library was previously designed by the Sooter Laboratory at WVU, and designated RMW.N34 [25]. The library consists of two 23-base constant regions for primer attachment during polymerase chain reaction (PCR), flanked by a 34-base random region, and it was commercially synthesized (Eurofins MWG Operon; Huntsville, AL, USA). Twelve rounds of SELEX were performed to identify ssDNA molecules that bound to fipronil (Table 1, Figure 6).

Fipronil (AccuStandard; New Haven, CT, USA) at a concentration of 42 µM in phosphate buffer saline (PBS)/20% acetone was covalently immobilized to carboxylic acid-coated magnetic beads (immobilizing substrate) (Dynabeads M-270 Carboxylic Acid, Life Technologies; Grand Island, NY, USA) via an amidation reaction using *N*-hydroxysulfonyl succinimide (sulfo-NHS) (Pierce; Rockford, IL, USA) and 1-ethyl-3-(3-dimethylaminopropyl) (EDC) (Pierce; Rockford, IL, USA) according to manufacturer’s protocol. After the conjugation reaction, beads were washed with phosphate buffer solution three times to remove unreacted fipronil. Unreacted carboxyl groups on the beads were quenched with 1× selection buffer (100 mM sodium chloride, 20 mM Tris-HCl, and 2 mM magnesium chloride). This provided the immobilized target (IT).

For positive rounds, 6.7 µL of IT was incubated with the ssDNA library in 500 µL of selection buffer (100 mM sodium chloride, 20 mM Tris-HCl, and 2 mM magnesium chloride; 1× selection buffer, SB) at room temperature with slow rotation. IT-bound ssDNA molecules were retrieved from the solution by magnetic separation, washed three times with 500 µL of 1× selection buffer, and resuspended in 100 µL of 1× selection buffer. This served as the PCR amplification template. The PCR conditions were as follows: bound ssDNA, 400 nM forward and biotinylated reverse RMW.N34 primers (Eurofins MWG Operon; Huntsville, AL, USA) (forward primer sequence: 5′-TGTACCGTCTGAGCGATTCGTAC-3′, biotinylated reverse primer sequence: 5′-Biotin-GCACTCCTTAACACTGACTGGCT-3′), 250 µM deoxynucleotide triphosphates, 1× GoTaq Reaction Buffer (Promega; Madison, WI, USA), 3.5 units *Taq*, and MilliQ pure water. Thermal cycling conditions were as follows: denature at 95 °C for 5 min, cycle at 95 °C for 1 min, 63 °C for 45 s, and 72 °C for 1 min; and final extension temperature at 72 °C for 7 min [24,25,26]. Large scale 4 mL PCR was carried out after each positive and negative selection round. This selection procedure for immobilized fipronil was performed for Rounds 1–6, each with decreasing incubation time.

PCR product with amplified dsDNA was purified with the IBI purification kit (IBI Scientific; Peosta, IA, USA) according to manufacturer’s protocol. Post-purification process includes single strand separation and ethanol precipitation of the forward strand DNA. These procedures were performed identically to as previously described [24,25,26].

For negative selection rounds, metabolites of fipronil (AccuStandard; New Haven, CT, USA), ethiprole (Sigma; St. Louis, MO, USA), and BSA were conjugated to carboxylic acid-coated magnetic beads as described above and served as immobilized negative targets. The selection process for negative Rounds 2–6 were performed similarly to positive Rounds 1–6, but unbound ssDNA molecules were instead retrieved and amplified.

Free fipronil was introduced in Round 7 positive and used for competitive elution. Competitive elution with the free target was performed to ensure the library does not bind to the immobilizing substrate, or the target only when it is immobilized. The incubation process was performed as positive Rounds 1–6, but free fipronil at a concentration of 500 µM in 1× selection buffer/0.5% methanol was used to elute ssDNA molecules bound to IT. The solution containing ssDNA bound to free fipronil was retrieved, and served as PCR template. The process was performed for positive Rounds 7–12, each with decreasing incubation time and free fipronil concentrations. For negative Rounds 7–11, similar competitive elutions were performed with free negative targets in solution. However, beads were retained, resuspended in selection buffer, and ssDNA bound to IT were amplified by PCR.

### 3.2. Cloning and Sequencing of the Fipronil-Binding MREs

The ssDNA library of post Rounds 3 negative, 6 negative, 9 negative and 12 positive, were cloned into competent *E. coli* cells, and plasmid DNA was extracted and analyzed for the enrichment of consensus binding sequences. These procedures were performed identically to as previously described [24,25,26]. A total of thirty to sixty randomly selected sequences from each listed round was analyzed.

### 3.3. Fipronil-Binding MREs Binding Assays in Selection Buffer

One candidate sequence designated as R12.51 from the round 12 sequences was selected for further characterization. The Mfold DNA web server was used for R12.51 secondary structure prediction with parameter settings at the ionic conditions of 1× selection buffer at 25 °C [27]. R12.51 was commercially synthesized with a 5′ FAM modification for the use of fluorescence saturation plate-based binding assay (Eurofins MWG Operon; Huntsville, AL, USA).

Fluorescence saturation plated-based binding assay was modified from previously described bead-based binding assays [24,25,26]. Concentrations of 0, 10, 20, 40, 60, 100, 500, and 1000 nM MRE were used in saturation binding assays. A 100 µL sample of 10 µM Fipronil in phosphate buffer solution was added to wells of a maleic anhydride activated 96-well microplate (Pierce; Rockford, IL, USA) and incubated with shaking at room temperature overnight. Following this preparation, each well was then blocked and washed with 200 µL of 1× selection buffer for 3 times, 10 min for each wash. FAM-labeled MRE of each concentration in a 100 µL total volume of 1× selection buffer was incubated with each well for five minutes. Unbound MRE was removed and each well was washed five times with 200 µL of 1× selection buffer, then 0.1 M of sodium hydroxide was added to each well to elute bound MRE. Eluted ssDNA was placed in a 96-well microplate and measured in a FLx800 microplate reader with excitation at 490 nm and emission at 520 nm (Biotek US, Winooski, VT, USA). All fluorescent readings on the plate were normalized to 100 µL of a 1 nM FAM-MRE in selection buffer with 0.1 M of sodium hydroxide. For each concentration set, a negative control was performed with wells containing no fipronil. Each set of concentrations were performed in triplicate. Three independent assays were performed as described above. GraphPad Prism 3 (GraphPad Software; La Jolla, CA, USA) was utilized to analyze the data and determine the dissociation constant (*K_d_*) of the MRE using nonlinear regression analysis and fit as previously described [24,25,26] Additional binding assay with concentrations of 0.5, 1, 5, 10, 15, 20, 40, 60, 100, 500, 1000, 2000, 5000, and 10,000 nM MRE were used. Assay was performed as described above. GraphPad Prism 3 was utilized to analyze the data and determine the dissociation constant (*K_d_*) of the MRE using nonlinear regression analysis and fit as described above, and the goodness of fit was determined with nonlinear regression sigmoidal analysis.

To determine the binding of R12.51 to negative targets used in the selection, fipronil was immobilized and microplate wells were washed and blocked exactly as described above. A 100 µL sample of 500 nM FAM-labeled MRE was added to each well for five minutes. Unbound MRE was removed and each well was washed as described above. Then, each well was incubated with 100 µL of 1 µM of the following in 1× selection buffer: fipronil sulfone, fipronil sulfide, fipronil desulfinyl, ethiprole, atrazine, propanil, malathion, BSA and in 1× selection buffer only. An incubation without MRE added and with fipronil as the eluent served as a normalization control. Each eluent solution was first incubated with the immobilized target for fifteen minutes, and then recovered. As described above, the solution was placed in a 96-well plate and the fluorescence was measured. Data were normalized to an internal fluorescent standard of 1nM FAM-labeled MRE and then the no-MRE control was subtracted as previously described [24,25,26]. Each set of specific binding studies were performed in triplicate. Data was averaged and standard deviation was calculated. A one-tailed student *t*-test was used to determine the statistical significance in difference of the means (*p* < 0.05).

### 3.4. Fipronil Cross-Binding Assay in River Water

To determine the binding of R12.51 to negative targets in river water, the experiment was performed similar to cross-binding assays performed in 1× selection buffer with minor modifications. River water was collected from the Susquehanna River at GPS coordinates 41°15′03.0′′ N 75°53′04.8′′ W. River water was first decanted to remove visible debris. It was then push-filtered through a 0.2 micron filter paper. An equal volume of 2× selection buffer was mixed with the prepared river water. Fipronil and all the negative targets were prepared in river water/buffer mix at 200 nM. Procedures and data analysis were performed as described in Section 3.3.

### 3.5. Fipronil Detection Assay in River Water

A proof-of-principle plate-based detection assay utilizing R12.51 was developed and modified from a previously described assay [48]. Fipronil was immobilized and microplate wells were washed and blocked exactly as described in Section 3.3. A 100 µL sample of 500 nM FAM-labeled MRE was added to each well for five minutes. Unbound MRE was removed and each well was washed as described above. Concentrations of 0, 5, 10, 25, 50, 100, 200, and 500 nM fipronil were used in detection assays. Varying concentrations of fipronil were spiked into the river water/buffer mix and subsequently added to each prepared well for fifteen minutes. The solution of each well was retrieved and fluorescence measurements were recorded as described in Section 3.3. Negative controls were wells without FAM-MRE and wells without immobilized fipronil. Three parallel experiments were performed. GraphPad Prism 3 was utilized to analyze the data and determine the *R*^2^ value with linear regression analysis. The limit of detection (LOD) was calculated as described by Armbruster et al. [41].

## 4. Conclusions

This study utilized a variant of SELEX methodology to identify a novel, broad-spectrum molecular recognition element that binds strongly to fipronil, its major metabolites and a structurally related pesticide. The MRE binds with a nanomolar equilibrium dissociation constant and is selective over structurally unrelated herbicides and pesticides that are found in the same environments as fipronil. Additionally, a proof-of-principle, plate-based fluorescent detection assay was developed and successfully used to detect fipronil in artificially contaminated river water samples. The results show the potential of utilizing this ssDNA MRE in biosensing applications, such as those described by Madianos et al. [46].

## Figures and Tables

**Table 1:** Systematic evolution of ligands by exponential enrichments (SELEX) scheme for fipronil-specific molecular recognition element (MRE) selection.

Round	Positive Selection	Time	Negative Selection	Time
1	Immobilized Target (IT)	48 h	Immobilizing substrate (IS)	24 h
2	IT	22 h	Immobilized Negative Target (INT) Fipronil Sulfide	24 h
3	IT	16 h	INT Fipronil Desulfinyl	24 h
4	IT	11 h	INT Fipronil Sulfone	24 h
5	IT	6.5 h	INT Ethiprole	24 h
6	IT	3 h	IT/ 1 mM of BSA Free Elution (FE)	3 h/ 24 h
7	IT/ Competitive Elution with 500 µM free Fipronil	3 h/ 1 h	IT/ 1 µM of Atrazine FE/1 µM of Propanil FE	3 h/ 24 h/ 24 h
8	IT/ Competitive Elution with 500 µM free Fipronil	1 h/ 30 min	IT/ 1 µM of Fipronil Sulfone FE/ 1 µM of Fipronil Desulfinyl FE	3 h/ 24 h/ 24 h
9	IT/ Competitive Elution with 100 µM free Fipronil	15 min/ 5 min	IT/ 1 µM of Fipronil Sulfide FE	15 min/ 15 min
10	IT/ Competitive Elution with 10 µM free Fipronil	1 min/ 1 min	IT/ 1 µM of Ethiprole Free Elution	1 min/ 1 min
11	IT/ Competitive Elution with 1 µM free Fipronil	Immediate/ Immediate	IT/ 1 mM of BSA FE/ 1 µM of Malathion FE	1 min/ 6 h/ 30 min
12	IT/ Competitive Elution with 100 nM free Fipronil	Immediate/ Immediate	-	

In vitro selection performed for identifying fipronil-specific MRE. Immobilized target (IT) is fipronil conjugated to magnetic beads. Immobilized negative target (INT) is negative targets conjugated to magnetic beads. BSA is the abbreviation for bovine serum albumin. FE is the abbreviation for free elution. Times listed are incubation times in hours (h), minutes (min) or immediate (few seconds).

**Table 2:** Cross-reactivity data of R12.51 ssDNA MRE in selection buffer.

Target	Normalized Average Fluorescence	Standard Deviation	*p*-Value	Selective Ratio
Fipronil	2.413	0.194	-	-
Fipronil Sulfone	2.248	0.388	0.279	1.07
Fipronil Sulfide	2.726	0.460	0.169	0.89
Fipronil Desulfinyl	2.088	0.623	0.219	1.16
Ethiprole	2.185	0.217	0.124	1.10
Atrazine	1.963	0.227	0.030	1.23
Propanil	1.587	0.243	0.005	1.52
Malathion	2.048	0.184	0.039	1.18
BSA	8.687	0.360	5.99 × 10^−6^	0.28
Selection Buffer (No Targets)	0.247	0.214	1.02 × 10^−4^	9.74

For each negative target (1 µM), normalized average fluorescence is given with 1× standard deviation. Each set of experiments was performed in triplicate. A one tailed student’s *t*-test was performed to calculate the *p*-value between fipronil and the cross-binding targets. The selectivity ratio represents the normalized average fluorescence for fipronil divided by the normalized average fluoresce for the cross-binding target, indicating R12.51’s level of preference for fipronil compared to the cross-binding target.

**Table 3:** Cross-reactivity data of R12.51 ssDNA MRE in river water/buffer mix.

Target	Normalized Average Fluorescence	Standard Deviation	*p*-Value	Selective Ratio
Fipronil	3.863	0.090	-	-
Fipronil Sulfone	2.863	0.180	4.98 × 10^−4^	1.34
Fipronil Sulfide	4.333	0.944	2.19 × 10^−1^	0.89
Fipronil Desulfinyl	2.902	0.245	15.5 × 10^−3^	1.33
Ethiprole	3.157	0.148	1.06 × 10^−3^	1.22
Atrazine	0.804	0.302	3.66 × 10^−5^	4.80
Propanil	0.764	0.509	2.44 × 10^−4^	5.05
Malathion	0.725	0.355	5.98 × 10^−5^	5.32
BSA	20.41	1.118	6.95 × 10^−6^	0.19
River water/Buffer Mix (No Targets)	−0.549	1.779	6.37 × 10^−3^	−7.035

For each negative target (200 nM), normalized average fluorescence is given with 1× standard deviation. Each set of experiments was performed in triplicate. A one tailed student’s *t*-test was performed to calculate the *p*-value between fipronil and cross-binding targets. The selectivity ratio represents the normalized average fluorescence for fipronil divided by the normalized average fluoresce for the cross-binding target, indicating R12.51’s level of preference for fipronil compared to the cross-binding target.

## Abbreviations

MRE	Molecular Recognition Element
BSA	Bovine Serum Albumin
IS	Immobilizing Substrate
IT	Immobilized Target
LOD	Limit of Detection
